# The Dentistry of To-Morrow1Read in the Section on Stomatology of the American Medical Association, at the fifty-sixth annual session, July, 1905, and republished by consent of the Journal of the American Medical Association.

**Published:** 1905-11

**Authors:** Harry P. Carlton

**Affiliations:** San Francisco, Cal.


### THE DENTISTRY OF TO-MORROW.[1]

BY HARRY P. CARLTON, D.D.S., SAN FRANCISCO, CAL.

Several years ago I had the opportunity to read before the Odontographic Society of Chicago a paper on "University Train-

---

[1] Read in the Section on Stomatology of the American Medical Association, at the fifty-sixth annual session, July, 1905, and republished by consent of the Journal of the American Medical Association.

ing and Dental Education," in which I took the ground that dentistry would only be a full profession and recognized as such when the character of the men who comprised it brought its recognition up to what it should be; that as this increase in dignity and professional status must come from within the body of the profession itself, a high grade of young men must be added to its ranks. I outlined the demands of the day and the way to meet those demands, making a plea for better educated material with which to begin our dental training, claiming that with these superior men we could shut the doors of our profession against the trifler and the tinker, and reserve the training of our schools for those who brought to the work the instincts, traditions, and outlook of the scholar; the result, real doctors of dental surgery.

I look on this occasion and this paper as but another chance to express my thoughts, to take another step forward, and to prophesy what seems to me the inevitable outcome. If dentistry is to continue to advance, along what lines must it go? It is pretty well conceded that mechanically we are close to the top notch, and only moderate advance can be looked for in that direction. The advance must then be along scientific lines, higher education, and the encouragement of research and investigation.

Dentistry is a specialty of medicine, but the only specialty not universally requiring the degree of doctor of medicine. Indeed, dentistry should be one of the most important branches or specialties of medicine, for in the mouth begin all those processes of nutrition and metabolism which of recent years have attracted so much attention. Thus far neither the physician nor the dentist has given enough scientific study to this special area of the human anatomy, and neither knows what the other has been doing or learning or is doing and learning.

Medicine and dentistry, though cognate sciences, have always been separately studied. This dissociation is now seen to be a great error, due not alone to the fact that the relation of the mouth and teeth to the rest of the anatomy was not understood, but also because only the mechanical aspect probably appealed to those who first considered the matter; no doubt dentistry had its beginning in the pulling of a tooth. This conception of dentistry as an entity and not as an integral part of medicine has made it an art, possibly a trade, rather than a science. A correct appreciation of physiology, pathology, and bacteriology—physiology representing the normal relation between the mouth and the rest of the alimentary canal; pathology showing the relation between the deranged functions of the mouth and abnormal conditions of other portions of the alimentary system; and bacteriology as an outgrowth of the study of pathology, showing the causes of the pathologic conditions of the mouth—necessitates an entirely different attitude, and is rapidly bringing the child, dentistry, back to its real parent, medicine. It has been said that "Dental science has brought the diseases of the mouth, jaws, and teeth so obviously under the domain of general pathology, that somatic problems elsewhere presented in the body are best and easiest studied in the mouth."

The opportunities of modern dentistry are so new and vast that not only is more manipulative skill demanded, but ampler education, more insight, more sagacity, faculties to whose development nature and elaborate training must both contribute. And this training must be given under the guidance of scientific teachers, non-practitioners, men of productive scholarship, men who devote their lives to the special work of teaching the various subjects which are embodied in a medical education, instead of by those who give instruction during the intervals of a busy practice.

There are new duties calling for a high degree of disciplined intelligence; to quote a recent article, "The knowledge of life and disease represented by the average D.D.S. degree is certainly deplorably deficient." How are we to remedy this? Give our young men a thorough medical training. Dentists are specialists only in their own handiwork, but the groundwork is uniform, and the demand for individualized education emphasizes the whole difference in our chosen tasks and ignores the great similarities. The technic of our branch of the profession, then, appears only as a small variation of the work in which we all share. The higher the level on which professional specialization begins, the more effective it is. And, again, "the higher the profession, the more nearly is the whole man working in every act, and the more, therefore, is a broad general education necessary."

How well I remember Professor Joseph Le Conte's likening of the modern system of education to the great sequoia, in which the primary and secondary schools were the rootlets and roots, the college culture courses the great bole, and the coming out from it at various altitudes as specializing branches, were the group of professional schools that make up the university. The trunk thus dividing grew higher and smaller, but persisted as the principal member all the way, until the topmost reach of pure culture was highest of all. Specializing without culture he likened to the low-branching bush, with no hope of reaching any fair height, and that the great branch of this tree of knowledge that deals with the study and care of the human body should spring from the main trunk after a considerable height of culture, at least that of the college curriculum, which Dr. Le Conte likened to the unbranching trunk, was the contention made in the former paper already mentioned. Without this no standing among the learned professions can be had at all. The half-light of the forest depth cannot be grown out of at any less altitude.

It is equally clear, also, that the great branch, the study of the human frame I have spoken of, must go out from the culture trunk of the university tree as a whole. The least consideration of its logical relations to the general culture and to other branches of knowledge will show the justness of this statement. And, having thus gone out from the main trunk, there is plenty of room for a considerable outward and upward reach of this great branch before it begins to subdivide into the various specialties, as the care of the various portions of the human body. So viewed, the mouth has as much relation to medical science, as a whole, as has the care of the eye, the ear, or, indeed, any other organ or group of organs.

He would be a superficial reasoner who could deny the great culture value of the medical course. Dr. Le Conte himself found it a firm foundation in its hold on physics and chemistry and a whole group of other sciences, and yet more in its cultivation of the habits of scientific observation of facts and logical reasoning from them for the stately structure of his own scientific attainments. The man who has taken it is a doctor, with none of the weakening qualifications of D.D.S., connoting a lesser scientific rank.

Most dental schools of the present time are at the mercy of the demands of the day and age, and their students are the product of the surroundings, not the choice of the school. An ideal professional school would be one that exists for its work alone, for scientific investigation and practical instruction not given by busy wage earners.

The dentist of to-morrow cannot be the same sort of man as the dentist of the past, whose own little sphere has been bounded by thirty-two teeth. He must be the carefully educated student in the medical branches, plus the dental specialties, and you can only make him so by following the same educational plan as in preparing the doctor of medicine. Without the requisite medical education, the ordinary mechanically expert dentist is not competent to practise his profession—he can only exercise a mechanical dexterity, but with the proper education he not only exercises the same dexterity, but he has professional ability.

The mouth is a fairly good barometer of the whole system, but your merely mechanical man, as contrasted with the dental specialist in medicine, is not competent to read it, and he does not. Why? How can any man untrained in medical science understand the problems presented to him in the condition of the mouth? We all know how exceedingly often some affection of the mouth or teeth is but an indication of a general systemic disturbance of metabolism; and, on the contrary, how frequently we see a faulty dental anatomy causing nutritive derangements which are puzzling to the physician who is ignorant of the large part played by the mouth in general alimentation. These things cannot be comprehended by the merely mechanically trained dentist, as distinguished from the dental specialist, any more than they are intelligible to the physician whose training has omitted all reference to the mouth as a vital part of the alimentary canal. He who would in the future confine his professional work to conditions involving the mouth and teeth—the dental specialist—must be a broad diagnostician; he must know the relation of the mouth to the general system, and he must interpret what he sees in the mouth in the light of his general medical education.

To bring about this condition—a condition which must surely come with the passage of time—the same preliminary training in the academic sciences will be required, whether the student intends to practise general medicine or surgery, or to specialize along the lines of ophthalmology, dermatology, orthopædic surgery, or dentistry.

The same general medical education must also be required, no matter to what special region of the anatomy nor to what special line of work the student intends to confine himself eventually.

What will be the results of this medical education? When this is accomplished what is going to become of your dentist? Practical life demands a division of labor; therefore, the specializing of the individual. There will be a division of the product just as in general medicine, and the natural ability of the individual will show itself. Some are going on plugging teeth, but better able, not less equipped for that work, for this higher education is not a question alone of preparing great men for great things; it must also prepare little men for greater things than would otherwise have been possible. Another lot are going to take up broad scientific work and research—not merely the mouth, but the mouth as merely a part, and the world will be spared from lots of trouble. But he who takes up the mechanical side of dental medicine has a broad and an inviting field of work before him. Thus far the orthopædic surgeon and the ophthalmologist who confines himself to refraction are most highly skilled mechanical specialists in medicine, but the medical dentist of the future will stand with them.

Consider the remarkably high degree of mechanical skill developed in the proper adjustment of a plaster jacket! Think of the tremendously complex Wullstein apparatus, devised for this purpose, and consider a man with no mechanical skill, in spite of any amount of medical education, attempting to apply such a jacket!

Dentistry, as well as orthopædic surgery, requires this highest kind of mechanical ability—take the apparatus used in orthodontia, for example; its construction and application call for the best mechanical skill, but they need back of that the academic training, or they do not reach the elevation. The real professional man should possess the resources of a highly developed, reasoning faculty, which comes only after years of systematic pursuit of what might be called higher or university study. It is this broad mental training that makes better dentists and better men. We are not only professional wage-earners—we live for our friends and our nation; we are in contact with nature and science, with art and literature; we shape our town and our time. Let us, then, provide the kind of men who know how to think.

Imagine a profession of mere operators; professional men! Not so; mechanical artisans. The hope of the profession lies not in fitting a system of instruction to the capacity of unpromising students, not in "substituting intellectual milk for intellectual meat,—aye, even in giving this milk in small quantity because of the puniness of the babes; but in choosing students equal to the requirements of an advanced method of instruction and substituting capable men for the class that now fill our colleges."

Turning out men with broad, well-balanced minds, with the faculty of judgment, strengthened by the mastery of principles rather than the acquisition of information. So much of the groundwork of medicine and dentistry is held in common that it is a wasteful folly to teach it in separate schools rather than in one well-equipped and well-manned institution. The cutting out of this unnecessary duplication of work is the spirit of the age and the method of much that we call our modern advance.

To summarize this point: The things that are purely groundwork, physics, inorganic chemistry, and all the branches that make for the building up of the man able to see clearly, to imagine vividly, and to reason correctly, as Dr. Henry Van Dyke puts it—these should be given in college. Then, all the studies necessary to the understanding of the human anatomy, physiology, and hygiene should be taken in a medical course; then, and then alone, is it time to specialize on oral surgery to make a dentist. It used to be said of Cuvier that, given a single bone, he could construct a whole animal, but he never arrived at that point by the study of one kind of bones only. Rather it is the method of to-day to study the whole skeleton, yes, and all the other parts of an animal as well, in order to understand one bone.

"Great truths approach slowly and dwell a long time with small minorities." Progress is the law of this American race, which stands for all that is best; truly it is a race which knows no rest. Was it not Macaulay who said, "A standing point which was yesterday invisible is the goal to-day and will be the starting post of to-morrow"?

DISCUSSION.

Dr. F. L. Platt, San Francisco, said that a degree from a university is certainly commendable and necessary, but he thinks that it is hardly all that is required. A large part of dentistry is mechanical and must necessarily continue to be so. He believes that in addition to a university degree, if a young man is going to study dentistry, he should also attend a school of manual training. Dr. Platt has noticed that the students who have had some manual training do superior work. There must be a combination of these two kinds of training before one can become a good dentist. After a man has gained a knowledge of chemistry, physics, bacteriology, and therapeutics, his training should be largely clinical. The greater part of dentistry can be taught by clinics, and Dr. Platt believes that each member of a class should be required to demonstrate his ability in the presence of others, and should not wait to learn to do this after he has acquired a degree. Experience in teaching operative technic has convinced Dr. Platt that so far as operative work is concerned, clinical instruction is far ahead of the lecture. He does not believe that dental colleges should be private corporations. Schools of law, medicine, theology, and dentistry should be integral parts of State universities, with funds provided by the State and faculties paid by the State. At the end of the first six months, if a student does not display aptitude for the work, he should be advised to take up something else. Dr. Platt agreed with Dr. Carlton that a high school education is not sufficient. Dr. Platt attended a pedagogic meeting a year ago, and heard many people speak who were in favor of a four years' course in dentistry, but did not hear a single sound argument in its favor. He thinks that the course should be at least nine months a year and four years if necessary, but to make the course four years at the expense of cutting down the length of each year, is not advancing. Students who have five months' vacation will not graduate from college as well equipped as students who have only three months' vacation. Some argue that the students get so tired they need five months' vacation. Others argue that students ought to have five months to work to earn money to finish their course. Neither of these arguments is worth considering. If the course is to be lengthened, let it be made four years of nine months each. A plan could be adopted requiring a degree in arts and letters for admission, as well as a manual school training, and then three years of good, earnest work would turn out good dentists.

Dr. M. L. Rhein, New York, said that possibly the colleges that have a six months' course are in the South, and they would have a six months' course whether it was three years or six. It has always seemed difficult to get Southern colleges to give any lengthy term. The tendency in the Northeast has been to increase the teaching term whatever the number of years. Dr. Rhein thinks the basis for dental education should be absolutely as high as that of medical education, and until that standard is reached dentists are below their true standard, and that is the only thing that keeps dentistry from being recognized as it should. It is the one thing that keeps students of the proper caliber from taking up this specialty. They do not care to place themselves on a lower plane than any of the other branches of medicine. This section should strive to make the requirements of dental education as high as that required for medicine, and to keep them up to that standard. Dr. Rhein thinks it impossible for a man to practise this branch of medicine without being thoroughly grounded in general medicine. The groundwork of medicine is recognized as being not only of value, but of necessity, and while dentistry is a technical work, and while a technical school is of the utmost importance, without the scientific knowledge of medicine the dentist is at a loss to properly apply his technical knowledge.

Dr. A. H. Levings, Milwaukee, said that there are many subjects in the medical curriculum which would be of no use to the dentist, though a dentist must have some knowledge of the fundamental branches, such as chemistry, pathology, bacteriology, histology, and therapeutics. He must have some knowledge of surgery and perhaps a smattering of medicine, but Dr. Levings thinks that the study of neurology, gynæcology, obstetrics, dermatology, otology, and allied subjects would be of no benefit to the dentist commensurate with the time spent in study. If the coming dentist were required to take a full course in medicine, he would have to spend an extra year in mechanical work, because the medical student's time is fully occupied. He cannot complete the course of to-day in less than four years of eight or nine months each. Dr. Levings said that he has heard many say that the requirements should be put so high that but few dental students could meet them. The only consideration should be what is best for these prospective dental students. Dr. Levings appreciates as much as any one that the more culture, refinement, education, and mental training an individual has, the higher he can rise in his profession, and the more easily he can maintain himself, but it is not within the possibility of every prospective dental student or every prospective medical student to secure the standard. Such a course as is given now in dental colleges will train any man who has a high school education so that he can master all the problems pertaining to either dentistry or medicine. Those who have the time and money may take a medical and a dental degree, and before this an A.B. degree, and polish themselves as much as possible.

Dr. M. I. Schamberg, Philadelphia, said that it may take some time before dentists will reach a higher standard than that existing at the present time. To his way of thinking, there are two things of prominent importance in taking up this subject: one is the raising of the standard of the profession, and the second, which he considers even more important, is the placing of such men in the dental world as are able to render the best possible service to humanity. The financial status of the dental college should be absolutely ignored. It may be that the work of the stomatologist and the dentist will ultimately become separated before arriving at the desired goal. If that be the case, it will probably be best for humanity at large. Dr. Schamberg would prefer to see the various dental institutions under the control of State universities, so that the financial side of the question would not enter into it so much as the educational.

Dr. G. V. I. Brown, Milwaukee, said that in this question of dental education a distinct advance of some kind is wanted. The purpose of Dr. Chittenden's paper is to fulfil the crowning act of a long life that has been given freely to the upraising of the standard, that before he dies something definite may be accomplished in the establishment of a higher standard of dental education. Dr. Brown said that he drew the resolution in the faculty association regarding the four-year term, and he has made more or less of a battle at different times for the four-year course. It is useless to discuss at this time the value of such a course, because for the time at least it has been decided to be inadvisable. He believes that the additional year could be secured with less hardship to the schools and with more likelihood of its being practical than any other advance. He believes every argument that has been made about the mechanical side of dentistry, and that since there cannot be a four-year course there ought to be higher entrance requirements, and that the course should be at least nine months, or as near that length as possible. Dr. Brown is connected with schools in the South and in the West. He sees both sides of the question, but at the present time no plan has been suggested which is practicable for meeting the situation. When some one presents a plan which will carry with it a distinct advance, so long as it is an advance which will enable the colleges of the South, West, and elsewhere to continue and to prosper, that plan will meet with approval, and when that time comes he has no doubt the examiners' and faculty associations will again be on an harmonious basis. Dr.

Brown believes in the value of having dental colleges under State control, but even under this condition it is not all smooth sailing by any means. At this time it seems nothing beneficial can be accomplished by discussion alone.

Dr. H. P. Carlton said that he has never yet written a paper of this character nor spoken his thoughts along this line, that the discussion did not at once turn to the question of courses and years. He wants to establish foundation courses and to leave the length of courses and curricula out of the question. He hopes to live to see it proved that the dentist of the future is going to be a medical man. The more a man gets in brain development the better dentist he will be. A man cannot be too broad and too scientifically trained to be a dentist.

